# 1-[3-(Naphthalen-1-yl)phen­yl]naphthal­ene^1^

**DOI:** 10.1107/S1600536813002407

**Published:** 2013-01-31

**Authors:** Manorama Tummala, Raj K. Dhar, Frank R. Fronczek, Steven F. Watkins

**Affiliations:** a Department of Chemistry, Louisiana State University, Baton Rouge, LA 70803-1804, USA

## Abstract

The title compound, C_26_H_18_, consists of a benzene ring with *meta*-substituted 1-naphthalene substituents, which are essentially planar (r.m.s. deviation = 0.039 and 0.027 Å). The conformation is mixed *syn*/*anti*, with equivalent torsion angles about the benzene–naphthalene bonds of 121.46 (11) and 51.58 (14)°.

## Related literature

For synthesis of the title compound, see: Woods *et al.* (1951 ▶). For similar structures, see Baker *et al.* (1990 ▶); Lin & Williams (1975 ▶); Bart (1968 ▶); Wolfenden *et al.* (2013 ▶). For *MM2* calculations, see: CambridgeSoft (2010 ▶).
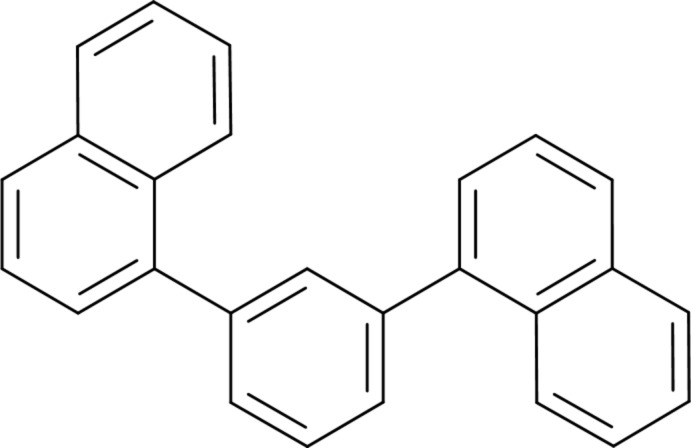


## Experimental

### 

#### Crystal data

C_26_H_18_
*M* *_r_* = 330.4
Triclinic, 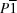

*a* = 7.6272 (1) Å
*b* = 10.8453 (2) Å
*c* = 11.8454 (2) Å
α = 106.0798 (8)°
β = 96.2976 (8)°
γ = 108.4307 (9)°
*V* = 872.05 (2) Å^3^
*Z* = 2
Mo *K*α radiation
μ = 0.07 mm^−1^
*T* = 100 K
0.28 × 0.22 × 0.15 mm

#### Data collection

Nonius KappaCCD diffractometer
Absorption correction: multi-scan (*SCALEPACK*; Otwinowski & Minor, 1997 ▶) *T*_min_ = 0.980, *T*_max_ = 0.989
11174 measured reflections
6272 independent reflections
4659 reflections with *I* > 2σ(*I*)
*R*_int_ = 0.025

#### Refinement

*R*[*F* ^2^ > 2σ(*F* ^2^)] = 0.050
*wR*(*F* ^2^) = 0.138
*S* = 1.05
6272 reflections
289 parameters
Only H-atom coordinates refined
Δρ_max_ = 0.36 e Å^−3^
Δρ_min_ = −0.25 e Å^−3^

### 

Data collection: *COLLECT* (Nonius, 2000 ▶); cell refinement: *SCALEPACK* (Otwinowski & Minor, 1997 ▶); data reduction: *DENZO* (Otwinowski & Minor, 1997 ▶) and *SCALEPACK*; program(s) used to solve structure: *SIR2002* (Burla *et al.*, 2003 ▶); program(s) used to refine structure: *SHELXL97* (Sheldrick, 2008 ▶); molecular graphics: *ORTEP-3 for Windows* (Farrugia, 2012 ▶); software used to prepare material for publication: *WinGX* (Farrugia, 2012 ▶).

## Supplementary Material

Crystal structure: contains datablock(s) global, I. DOI: 10.1107/S1600536813002407/tk5189sup1.cif

Structure factors: contains datablock(s) I. DOI: 10.1107/S1600536813002407/tk5189Isup2.hkl

Supplementary material file. DOI: 10.1107/S1600536813002407/tk5189Isup3.cml

Additional supplementary materials:  crystallographic information; 3D view; checkCIF report

## Figures and Tables

**Fig. 1.**
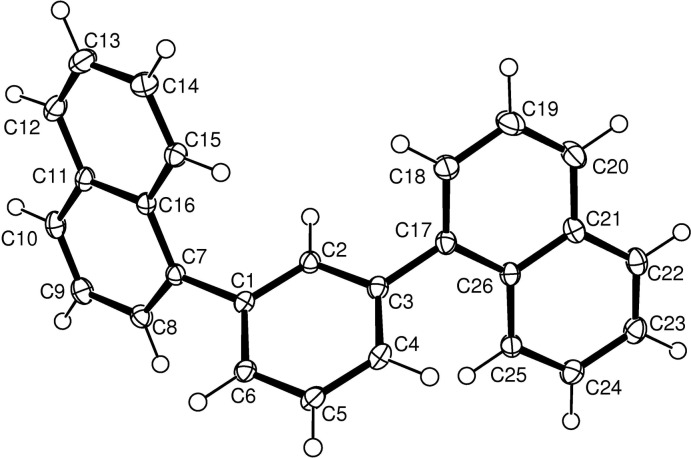
View of (I) (50% probability displacement ellipsoids)

## supplementary crystallographic information

### Comment

Although the structures of *p*-oligophenyls have been well investigated (Baker *et al.*, 1990, and references therein), there have been few reports of the conformational preferences of *m*-oligophenyls. Lin & Williams (1975) have reported the crystal structure of 1,3,5 triphenyl benzene, which serves as a model for *m*-polyphenyls. That structure has phenyl groups which are twisted about the benzene-benzene single bonds by torsion angles of +40.7, -37.2, and +36.1°. The crystal structure of one of the polymorphic forms of hexaphenyl benzene, reported by Bart (1968), also shows that the peripheral rings are twisted out of the central ring by about 25°. That molecule also exhibited out-of-plane distortion by bending of the exocyclic bonds. We have studied the structure of 1,3-bis(1-naphthyl)benzene for comparison of its conformation to the previous results.

Title compound I consists of a benzene ring with *meta*-substituted 1-naphthalenes. The benzene ring is planar (δ_r.m.s._ = 0.007 Å), as are the two naphthalenes (δ_r.m.s._ = 0.039 and 0.027 Å). *MM2* calculations of isolated models (CambridgeSoft, 2010) reveal six conformers of I with approximately equal energies. They differ by positive or negative torsions C2—C1—C7—C8 (A1) and C2—C3—C17—C18 (A2) from the three paradigmatic conformers *syn* (C_2v_, A1, A2 = 0, 0°), *anti* (C_2v_, 180, 180°), and mixed (C_s_, 180, 0°). The conformation of I is mixed (C_1_), with A1 = 121.46 (11)° and A2 = 51.58 (14)° (*MM2* yields angles of 149 and 35° for the global minimum energy conformation).

### Experimental

The crystal was prepared by refluxing di-(α-naphthyl)-cyclohexadiene with a Pd-charcoal mixture in *p*-cymene for 4–5 h. After filtration, the filtrate was steam distilled. The residue was extracted with ether and recrystallized from petroleum ether (Woods *et al.*, 1951).

### Refinement

All H atom positions were refined, but *U*_iso_(H) was set to 1.2*U*_eq_ of the attached C atom. C–H distances fall within the range 0.973 (14) - 1.031 (15) Å.

### Crystal data

**Table:**

C_26_H_18_	*Z* = 2
*M**_r_* = 330.4	*F*(000) = 348
Triclinic, *P*1	*D*_x_ = 1.258 Mg m^−^^3^
Hall symbol: -P 1	Melting point: 131.5(5) K
*a* = 7.6272 (1) Å	Mo *K*α radiation, λ = 0.71073 Å
*b* = 10.8453 (2) Å	Cell parameters from 5528 reflections
*c* = 11.8454 (2) Å	θ = 2.6–32.6°
α = 106.0798 (8)°	µ = 0.07 mm^−^^1^
β = 96.2976 (8)°	*T* = 100 K
γ = 108.4307 (9)°	Prism, colourless
*V* = 872.05 (2) Å^3^	0.28 × 0.22 × 0.15 mm

### Data collection

**Table:**

Nonius KappaCCD diffractometer	6272 independent reflections
Radiation source: fine-focus sealed tube	4659 reflections with *I* > 2σ(*I*)
Graphite monochromator	*R*_int_ = 0.025
Detector resolution: 9 pixels mm^-1^	θ_max_ = 32.6°, θ_min_ = 2.9°
φ and ω scans	*h* = −11→11
Absorption correction: multi-scan (*SCALEPACK*; Otwinowski & Minor, 1997)	*k* = −16→16
*T*_min_ = 0.980, *T*_max_ = 0.989	*l* = −17→17
11174 measured reflections	

### Refinement

**Table:**

Refinement on *F*^2^	Primary atom site location: structure-invariant direct methods
Least-squares matrix: full	Secondary atom site location: difference Fourier map
*R*[*F*^2^ > 2σ(*F*^2^)] = 0.050	Hydrogen site location: inferred from neighbouring sites
*wR*(*F*^2^) = 0.138	Only H-atom coordinates refined
*S* = 1.05	*w* = 1/[σ^2^(*F*_o_^2^) + (0.0681*P*)^2^ + 0.1782*P*] where *P* = (*F*_o_^2^ + 2*F*_c_^2^)/3
6272 reflections	(Δ/σ)_max_ = 0.001
289 parameters	Δρ_max_ = 0.36 e Å^−^^3^
0 restraints	Δρ_min_ = −0.25 e Å^−^^3^
0 constraints	

### Special details

**Table:**

Geometry. All e.s.d.'s (except the e.s.d. in the dihedral angle between two l.s. planes) are estimated using the full covariance matrix. The cell e.s.d.'s are taken into account individually in the estimation of e.s.d.'s in distances, angles and torsion angles; correlations between e.s.d.'s in cell parameters are only used when they are defined by crystal symmetry. An approximate (isotropic) treatment of cell e.s.d.'s is used for estimating e.s.d.'s involving l.s. planes.

### Fractional atomic coordinates and isotropic or equivalent isotropic displacement parameters (Å^2^)

**Table:**

	*x*	*y*	*z*	*U*_iso_*/*U*_eq_	
C1	0.82582 (14)	0.47008 (10)	0.81621 (9)	0.01484 (18)	
C2	0.69555 (14)	0.50351 (10)	0.74916 (9)	0.01559 (18)	
H2	0.5744 (18)	0.4321 (13)	0.7006 (12)	0.019*	
C3	0.73683 (14)	0.63687 (10)	0.74479 (9)	0.01583 (18)	
C4	0.91076 (15)	0.73893 (10)	0.81138 (9)	0.0186 (2)	
H4	0.9414 (19)	0.8349 (14)	0.8107 (13)	0.022*	
C5	1.04081 (15)	0.70701 (11)	0.87862 (9)	0.0192 (2)	
H5	1.159 (2)	0.7783 (14)	0.9277 (13)	0.023*	
C6	0.99993 (14)	0.57264 (10)	0.87979 (9)	0.01680 (19)	
H6	1.0961 (19)	0.5510 (13)	0.9275 (12)	0.02*	
C7	0.78015 (13)	0.32634 (10)	0.81691 (9)	0.01449 (18)	
C8	0.89726 (15)	0.25621 (11)	0.77886 (10)	0.01835 (19)	
H8	1.013 (2)	0.3032 (14)	0.7538 (13)	0.022*	
C9	0.85267 (16)	0.11697 (11)	0.77163 (10)	0.0213 (2)	
H9	0.939 (2)	0.0692 (15)	0.7408 (14)	0.026*	
C10	0.69203 (16)	0.04918 (10)	0.80376 (10)	0.0200 (2)	
H10	0.658 (2)	−0.0494 (14)	0.7972 (13)	0.024*	
C11	0.57133 (14)	0.11841 (10)	0.84745 (9)	0.01646 (19)	
C12	0.40719 (16)	0.05083 (11)	0.88491 (10)	0.0216 (2)	
H12	0.380 (2)	−0.0479 (15)	0.8764 (13)	0.026*	
C13	0.29543 (16)	0.11944 (12)	0.93179 (11)	0.0241 (2)	
H13	0.183 (2)	0.0731 (15)	0.9603 (13)	0.029*	
C14	0.34088 (16)	0.25987 (11)	0.94264 (10)	0.0217 (2)	
H14	0.261 (2)	0.3096 (14)	0.9777 (13)	0.026*	
C15	0.49569 (14)	0.32721 (10)	0.90471 (9)	0.01736 (19)	
H15	0.5279 (19)	0.4277 (14)	0.9140 (13)	0.021*	
C16	0.61575 (14)	0.25913 (9)	0.85565 (9)	0.01443 (18)	
C17	0.59275 (14)	0.66850 (10)	0.67417 (9)	0.01692 (19)	
C18	0.41114 (16)	0.63294 (12)	0.69381 (11)	0.0235 (2)	
H18	0.378 (2)	0.5841 (15)	0.7523 (14)	0.028*	
C19	0.27133 (17)	0.66524 (14)	0.63265 (12)	0.0287 (3)	
H19	0.139 (2)	0.6350 (16)	0.6469 (14)	0.034*	
C20	0.31553 (16)	0.73679 (13)	0.55434 (11)	0.0247 (2)	
H20	0.216 (2)	0.7599 (15)	0.5088 (14)	0.03*	
C21	0.49962 (15)	0.77453 (10)	0.53030 (9)	0.01804 (19)	
C22	0.54657 (16)	0.84667 (11)	0.44775 (9)	0.0201 (2)	
H22	0.4519 (19)	0.8779 (14)	0.4119 (13)	0.024*	
C23	0.71984 (16)	0.87506 (11)	0.41886 (10)	0.0220 (2)	
H23	0.751 (2)	0.9230 (14)	0.3557 (14)	0.026*	
C24	0.85601 (16)	0.83222 (11)	0.47117 (10)	0.0215 (2)	
H24	0.983 (2)	0.8506 (15)	0.4477 (13)	0.026*	
C25	0.81667 (15)	0.76545 (11)	0.55395 (10)	0.01813 (19)	
H25	0.9135 (19)	0.7354 (14)	0.5896 (13)	0.022*	
C26	0.63915 (14)	0.73646 (10)	0.58754 (9)	0.01580 (18)	

### Atomic displacement parameters (Å^2^)

**Table:**

	*U*^11^	*U*^22^	*U*^33^	*U*^12^	*U*^13^	*U*^23^
C1	0.0164 (4)	0.0144 (4)	0.0145 (4)	0.0052 (3)	0.0046 (3)	0.0059 (3)
C2	0.0164 (4)	0.0139 (4)	0.0158 (4)	0.0050 (3)	0.0026 (3)	0.0049 (3)
C3	0.0181 (4)	0.0162 (4)	0.0149 (4)	0.0068 (3)	0.0050 (3)	0.0065 (3)
C4	0.0229 (5)	0.0152 (4)	0.0176 (5)	0.0046 (4)	0.0059 (4)	0.0072 (4)
C5	0.0195 (5)	0.0178 (4)	0.0166 (5)	0.0007 (4)	0.0028 (4)	0.0074 (4)
C6	0.0160 (4)	0.0188 (4)	0.0152 (4)	0.0041 (3)	0.0030 (3)	0.0079 (3)
C7	0.0153 (4)	0.0141 (4)	0.0137 (4)	0.0051 (3)	0.0016 (3)	0.0048 (3)
C8	0.0185 (5)	0.0197 (4)	0.0188 (5)	0.0089 (4)	0.0047 (4)	0.0068 (4)
C9	0.0251 (5)	0.0205 (5)	0.0217 (5)	0.0134 (4)	0.0046 (4)	0.0062 (4)
C10	0.0262 (5)	0.0152 (4)	0.0192 (5)	0.0098 (4)	0.0019 (4)	0.0054 (4)
C11	0.0197 (5)	0.0131 (4)	0.0150 (4)	0.0044 (3)	0.0010 (3)	0.0050 (3)
C12	0.0237 (5)	0.0169 (4)	0.0217 (5)	0.0023 (4)	0.0036 (4)	0.0089 (4)
C13	0.0213 (5)	0.0239 (5)	0.0247 (5)	0.0024 (4)	0.0066 (4)	0.0106 (4)
C14	0.0205 (5)	0.0236 (5)	0.0218 (5)	0.0078 (4)	0.0076 (4)	0.0078 (4)
C15	0.0189 (5)	0.0163 (4)	0.0175 (5)	0.0067 (4)	0.0047 (4)	0.0059 (4)
C16	0.0158 (4)	0.0134 (4)	0.0134 (4)	0.0049 (3)	0.0014 (3)	0.0046 (3)
C17	0.0193 (4)	0.0163 (4)	0.0178 (5)	0.0086 (4)	0.0052 (4)	0.0066 (4)
C18	0.0218 (5)	0.0307 (5)	0.0265 (6)	0.0132 (4)	0.0112 (4)	0.0159 (5)
C19	0.0212 (5)	0.0429 (7)	0.0334 (6)	0.0174 (5)	0.0124 (5)	0.0205 (5)
C20	0.0235 (5)	0.0332 (6)	0.0253 (6)	0.0175 (5)	0.0068 (4)	0.0127 (5)
C21	0.0207 (5)	0.0182 (4)	0.0162 (4)	0.0100 (4)	0.0023 (4)	0.0044 (4)
C22	0.0250 (5)	0.0188 (4)	0.0165 (5)	0.0099 (4)	0.0000 (4)	0.0049 (4)
C23	0.0264 (5)	0.0209 (5)	0.0176 (5)	0.0062 (4)	0.0018 (4)	0.0087 (4)
C24	0.0219 (5)	0.0233 (5)	0.0208 (5)	0.0072 (4)	0.0060 (4)	0.0103 (4)
C25	0.0190 (5)	0.0191 (4)	0.0193 (5)	0.0086 (4)	0.0052 (4)	0.0087 (4)
C26	0.0183 (4)	0.0143 (4)	0.0158 (4)	0.0073 (3)	0.0037 (3)	0.0048 (3)

### Geometric parameters (Å, º)

**Table:**

C1—C6	1.3966 (14)	C13—C14	1.4157 (16)
C1—C2	1.4013 (13)	C13—H13	0.992 (15)
C1—C7	1.4886 (13)	C14—C15	1.3720 (15)
C2—C3	1.3966 (13)	C14—H14	0.988 (15)
C2—H2	0.984 (13)	C15—C16	1.4241 (14)
C3—C4	1.4019 (14)	C15—H15	1.010 (13)
C3—C17	1.4902 (14)	C17—C18	1.3799 (15)
C4—C5	1.3909 (15)	C17—C26	1.4337 (14)
C4—H4	0.995 (13)	C18—C19	1.4113 (16)
C5—C6	1.3949 (14)	C18—H18	0.990 (15)
C5—H5	0.973 (14)	C19—C20	1.3704 (17)
C6—H6	1.001 (13)	C19—H19	1.007 (16)
C7—C8	1.3811 (14)	C20—C21	1.4174 (16)
C7—C16	1.4309 (14)	C20—H20	1.016 (15)
C8—C9	1.4144 (15)	C21—C22	1.4212 (15)
C8—H8	0.991 (14)	C21—C26	1.4272 (13)
C9—C10	1.3690 (16)	C22—C23	1.3634 (16)
C9—H9	0.999 (15)	C22—H22	0.992 (14)
C10—C11	1.4161 (15)	C23—C24	1.4149 (15)
C10—H10	0.996 (14)	C23—H23	1.031 (15)
C11—C12	1.4223 (15)	C24—C25	1.3771 (14)
C11—C16	1.4273 (13)	C24—H24	1.010 (15)
C12—C13	1.3656 (17)	C25—C26	1.4173 (14)
C12—H12	0.998 (14)	C25—H25	0.995 (14)
			
C6—C1—C2	119.05 (9)	C15—C14—C13	120.27 (10)
C6—C1—C7	120.75 (9)	C15—C14—H14	119.9 (8)
C2—C1—C7	120.18 (8)	C13—C14—H14	119.8 (8)
C3—C2—C1	121.27 (9)	C14—C15—C16	121.25 (9)
C3—C2—H2	118.1 (7)	C14—C15—H15	119.6 (8)
C1—C2—H2	120.5 (7)	C16—C15—H15	119.1 (8)
C2—C3—C4	118.73 (9)	C15—C16—C11	118.13 (9)
C2—C3—C17	119.48 (9)	C15—C16—C7	122.94 (9)
C4—C3—C17	121.74 (9)	C11—C16—C7	118.92 (9)
C5—C4—C3	120.47 (9)	C18—C17—C26	119.33 (9)
C5—C4—H4	119.7 (8)	C18—C17—C3	119.14 (9)
C3—C4—H4	119.8 (8)	C26—C17—C3	121.53 (9)
C4—C5—C6	120.26 (9)	C17—C18—C19	121.38 (10)
C4—C5—H5	120.7 (8)	C17—C18—H18	119.4 (8)
C6—C5—H5	119.0 (8)	C19—C18—H18	119.3 (9)
C5—C6—C1	120.18 (9)	C20—C19—C18	120.19 (11)
C5—C6—H6	119.3 (8)	C20—C19—H19	120.4 (9)
C1—C6—H6	120.5 (7)	C18—C19—H19	119.4 (9)
C8—C7—C16	119.38 (9)	C19—C20—C21	120.53 (10)
C8—C7—C1	119.62 (9)	C19—C20—H20	121.2 (9)
C16—C7—C1	121.00 (8)	C21—C20—H20	118.2 (9)
C7—C8—C9	121.24 (10)	C20—C21—C22	121.15 (9)
C7—C8—H8	119.6 (8)	C20—C21—C26	119.53 (9)
C9—C8—H8	119.1 (8)	C22—C21—C26	119.29 (9)
C10—C9—C8	120.22 (10)	C23—C22—C21	121.06 (9)
C10—C9—H9	121.2 (8)	C23—C22—H22	119.8 (8)
C8—C9—H9	118.6 (8)	C21—C22—H22	119.1 (8)
C9—C10—C11	120.51 (9)	C22—C23—C24	119.94 (10)
C9—C10—H10	120.8 (8)	C22—C23—H23	120.9 (8)
C11—C10—H10	118.7 (8)	C24—C23—H23	119.1 (8)
C10—C11—C12	121.23 (9)	C25—C24—C23	120.38 (10)
C10—C11—C16	119.64 (9)	C25—C24—H24	119.7 (8)
C12—C11—C16	119.13 (9)	C23—C24—H24	119.9 (8)
C13—C12—C11	121.13 (10)	C24—C25—C26	121.10 (9)
C13—C12—H12	122.3 (8)	C24—C25—H25	119.5 (8)
C11—C12—H12	116.6 (8)	C26—C25—H25	119.4 (8)
C12—C13—C14	120.05 (10)	C25—C26—C21	118.13 (9)
C12—C13—H13	121.2 (9)	C25—C26—C17	123.00 (9)
C14—C13—H13	118.8 (8)	C21—C26—C17	118.85 (9)
			
C6—C1—C2—C3	0.09 (15)	C12—C11—C16—C7	−179.48 (9)
C7—C1—C2—C3	−178.50 (9)	C8—C7—C16—C15	175.10 (9)
C1—C2—C3—C4	−1.45 (15)	C1—C7—C16—C15	−5.35 (15)
C1—C2—C3—C17	−178.88 (9)	C8—C7—C16—C11	−3.52 (14)
C2—C3—C4—C5	1.18 (15)	C1—C7—C16—C11	176.03 (9)
C17—C3—C4—C5	178.55 (10)	C2—C3—C17—C18	51.58 (14)
C3—C4—C5—C6	0.46 (16)	C4—C3—C17—C18	−125.77 (12)
C4—C5—C6—C1	−1.86 (16)	C2—C3—C17—C26	−129.21 (11)
C2—C1—C6—C5	1.58 (15)	C4—C3—C17—C26	53.44 (14)
C7—C1—C6—C5	−179.85 (10)	C26—C17—C18—C19	−1.65 (17)
C6—C1—C7—C8	−57.09 (13)	C3—C17—C18—C19	177.57 (11)
C2—C1—C7—C8	121.46 (11)	C17—C18—C19—C20	−1.89 (19)
C6—C1—C7—C16	123.36 (11)	C18—C19—C20—C21	2.44 (19)
C2—C1—C7—C16	−58.08 (13)	C19—C20—C21—C22	178.91 (11)
C16—C7—C8—C9	3.27 (15)	C19—C20—C21—C26	0.53 (17)
C1—C7—C8—C9	−176.28 (9)	C20—C21—C22—C23	−175.72 (10)
C7—C8—C9—C10	−0.69 (16)	C26—C21—C22—C23	2.66 (15)
C8—C9—C10—C11	−1.62 (16)	C21—C22—C23—C24	0.14 (16)
C9—C10—C11—C12	−177.93 (10)	C22—C23—C24—C25	−1.92 (17)
C9—C10—C11—C16	1.29 (15)	C23—C24—C25—C26	0.83 (17)
C10—C11—C12—C13	177.18 (10)	C24—C25—C26—C21	1.96 (15)
C16—C11—C12—C13	−2.04 (16)	C24—C25—C26—C17	−179.34 (10)
C11—C12—C13—C14	0.68 (17)	C20—C21—C26—C25	174.76 (10)
C12—C13—C14—C15	0.87 (17)	C22—C21—C26—C25	−3.65 (14)
C13—C14—C15—C16	−1.04 (16)	C20—C21—C26—C17	−4.00 (14)
C14—C15—C16—C11	−0.33 (15)	C22—C21—C26—C17	177.59 (9)
C14—C15—C16—C7	−178.95 (10)	C18—C17—C26—C25	−174.15 (10)
C10—C11—C16—C15	−177.40 (9)	C3—C17—C26—C25	6.64 (15)
C12—C11—C16—C15	1.83 (14)	C18—C17—C26—C21	4.54 (15)
C10—C11—C16—C7	1.28 (14)	C3—C17—C26—C21	−174.67 (9)

## References

[bb1] Baker, K. N., Fratini, A. V. & Adams, W. W. (1990). *Polymer*, **31**, 1623–1631.

[bb2] Bart, J. C. J. (1968). *Acta Cryst.* B**24**, 1277–1287.

[bb3] Burla, M. C., Camalli, M., Carrozzini, B., Cascarano, G. L., Giacovazzo, C., Polidori, G. & Spagna, R. (2003). *J. Appl. Cryst.* **36**, 1103.

[bb4] CambridgeSoft (2010). *Chem3DPro.* CambridgeSoft Corporation, Cambridge, MA, USA.

[bb5] Farrugia, L. J. (2012). *J. Appl. Cryst.* **45**, 849–854.

[bb6] Lin, Y. C. & Williams, D. E. (1975). *Acta Cryst.* B**31**, 318–320.

[bb7] Nonius (2000). *COLLECT* Nonius BV, Delft, The Netherlands.

[bb8] Otwinowski, Z. & Minor, W. (1997). *Methods in Enzymology*, Vol. 276, *Macromolecular Crystallography*, Part A, edited by C. W. Carter Jr & R. M. Sweet, pp. 307–326. New York: Academic Press.

[bb9] Sheldrick, G. M. (2008). *Acta Cryst.* A**64**, 112–122. 10.1107/S0108767307043930 18156677

[bb11] Wolfenden, M. L., Dhar, R. K., Fronczek, F. R. & Watkins, S. F. (2013). *Acta Cryst.* E**69**, o308. 10.1107/S1600536813002390 PMC3569827 23424573

[bb10] Woods, G. F., Reed, F. T., Arthur, T. E. & Ezekiel, H. (1951). *J. Am. Chem. Soc.* **73**, 3854–3856.

